# Impact of Exposure to Natural and Built Environments on Positive and Negative Affect: A Systematic Review and Meta-Analysis

**DOI:** 10.3389/fpubh.2021.758457

**Published:** 2021-11-25

**Authors:** Wenfei Yao, Fei Chen, San Wang, Xiaofeng Zhang

**Affiliations:** ^1^Department of Landscape Architecture, College of Architecture and Urban Planning, Qingdao University of Technology, Qingdao, China; ^2^Innovation Institute for Sustainable Maritime Architecture Research and Technology (iSMART), Qingdao University of Technology, Qingdao, China

**Keywords:** natural environment, built environment, meta-analysis, positive affect (PA), negative affect (NA)

## Abstract

There is increasing evidence that the natural environment provides substantial benefits to human emotional well-being. The current study synthesized this body of research using the meta-analysis and assessed the positive and negative effects of exposure to both the natural and built environments. We searched four databases and 20 studies were included in the review. The meta-analysis results showed the most convincing evidence that exposure to the natural environment could increase positive affect (standardized mean difference, SMD = 0.61, 95% *CI* 0.41, 0.81) and decreased negative affect (SMD = −0.47, 95% *CI* −0.71, −0.24). However, there was extreme heterogeneity between the studies, and the risk of bias was high. According to the subgroup analysis, study region, study design, mean age of the sample, sample size, and type of natural and built environment were found to be important factors during exposure to the natural environment. The implications of these findings for the existing theory and research are discussed. These findings will help convince the health professionals and policymakers to encourage the residents to increase their time spent in the natural environment. These findings of this systematic review also suggested that the creation, maintenance, and enhancement of accessible greenspaces or existing natural environments may form part of a multidimensional approach to increasing emotional well-being of the local populations.

## Introduction

A considerable number of theoretical and empirical studies support the notion that natural exposure contributes to both physiological and psychological health (1–5). Exposure to the natural environment is associated with psychological health, such as reducing negative emotions and fatigue, increasing energy, improving attention, and increasing satisfaction and enjoyment (6). Especially with the quick development of urbanization, there is an increasing recognition that the natural environment is a potential buffer for poor mental health (7, 8).

Several existing theories demonstrate that the natural environment is beneficial to the mental health of the population. Two existing theories focus on the effects of the natural environment on human health. The stress reduction theory (9) proposed that the presence of nature brings with it an evolutionary response to safety and survival which produces positive emotions (10). In support of this theory, the empirical studies indicated lower physiological arousal, less negative affect, and higher positive affect in the participants exposed to the natural environment when compared with those exposed to the built environment (11–14). An alternative theory that concerns the effect of nature on well-being is the attention recovery theory (ART) (15). According to ART, fast-paced urban living leads to cognitive fatigue, which may manifest as difficulty concentrating, the higher levels of irritability, and negative affect. The natural environment provides a “soft fascination” that allows a person to pay attention effortlessly. Numerous studies have provided empirical support for the prediction of ART, with the subjects indicating better cognitive functioning and more positive emotions following exposure to the natural environment (16–19).

As the above literature indicates, ample empirical evidence suggests that exposure to the natural environment is associated with increased emotional well-being. However, few studies have provided a quantitative synthesis of effect of the natural environment on the positive and negative emotions, especially for comparison with built environments. The hypotheses predict that if the individuals are stressed, an encounter with most unthreatening natural environments will have a stress reduction or restorative influence, whereas many built environments will hamper recuperation (9). Generally, natural landscapes have a stronger positive health effect than the urban landscapes. The studies have found that the effects of urban landscapes on health are less positive and, in some cases, even negative (20). Our primary objective in this systematic review and meta-analysis was to quantify the effect of natural and built environments on one facet of psychological well-being, namely, emotional well-being. Positive and negative affect are the two key components of this (21), as their outcome gives the researchers and therapists information concerning the mental state of the participants and patients. We examined the positive and negative effects of exposure to natural and built environments to compare the strength of the influence of both the environments. The second objective of the current study was to identify any potential moderators (such as, mean age of the sample, study region, study design, and sample size) that impact the effect of exposure to the natural environment on emotional well-being. The third objective was to evaluate the quality of the available evidence and thus to provide comprehensive evidence regarding the effect of the natural environment on positive and negative affect, discovering and addressing the limitations of existing theory and research, which may provide an opportunity for fruitful future work in this area.

## Methods

### Search Strategy

The protocol was registered using PROSPERO (22). We conducted a systematic review and meta-analysis according to the Preferred Reporting Items for Systematic Reviews and Meta-Analysis (PRISMA) guidelines as shown in Supplementary Table 1 (23, 24). A specific research question was formulated according to the “Participants,” “Exposure,” “Comparator,” and “Outcomes” (PECO) framework. The focus of the present systematic review and meta-analysis was: “Is exposure to the natural environment associated with positive and negative affect?” We searched the electronic databases, such as Web of Science, PubMed, ScienceDirect, and PsychINFO. Our search strategies were based on the combinations of natural environment terms (“natural environment,” “urban green environments,” “urban park,” “urban forest,” and “therapeutic landscape”) and positive and negative emotion terms (“positive affect,” “positive emotion,” “negative emotion,” “negative affect,” and “well-being”). The detailed search strategy is shown in Supplementary Table 2. The reference sections of the obtained papers were examined for the additional studies, and a descendancy search was conducted for the studies that cited the obtained papers.

### Selection Criteria

The following *aprior* eligibility criteria were based on the PECO framework:

- **Participants:** any human adult population
- **Exposure:** exposure to the natural environment
- **Comparator:** exposure to the built environment
- **Outcomes:** positive and negative affect.

In terms of the populations considered, any adult population was eligible, regardless of the physical or mental health conditions. The studies involving children were excluded from the review (2). Exposure was defined as the placement of participants in direct physical or sensory contact with a real exposure environment, within the context of a randomized or non-randomized trial. The duration of exposure was not limited. The representations of an environment using virtual reality, photography, or video were not included (25–29). The environments were deemed as “natural” if they were defined by a high level of greenness and had not been extensively transformed by human activity. In contrast, a built environment was defined as a predominantly man-made environment with a low level of greenness or an indoor environment (10, 14). The studies in which the participants were engaged in more than two environments were eligible, however, only data from the natural and built environments were included in the meta-analysis. The studies had to include a comparison group (natural *vs*. built environment) and a self-report assessment of current emotional state that was administered following exposure to the natural and built conditions. The studies that did not include an emotion assessment and examined only cognitive and/or psychophysiological responses to nature were excluded.

The reviewers (XZ) initially screened titles and abstracts to remove obviously irrelevant articles, and then the two reviewers (WY and CF) independently screened all the full texts for eligibility. The discrepancies were resolved through discussion.

### Data Extraction

Two reviewers (WY and XZ) independently extracted the following information from each included study: the names of authors, publication year, location, study design, sample, environment type, exposure procedure, and effect measures. Data were extracted into a coding frame using Microsoft Excel, synthesized, and tabulated. We used the risk of bias tool employed by Twohig-Bennett and Jones (30) and Ogilvie et al. (31) (Supplementary Table 3) which was adapted for this purpose.

### Narrative Summary and Meta-Analysis

A narrative summary was compiled after a critical review of each study. All the studies were considered to be included in the meta-analysis. Mean, SD, and cell count (N) for all positive and negative affect the outcomes in each included study were extracted. In the first instance, data were extracted directly from the studies.

The studies that included the participants exposed to the natural environment only were excluded from the review (1, 32–34). Two studies had multiple natural environments: Tyrväinen et al. (35) investigated the psychological and physiological effects of short-term visits to city centers, urban parks, and woodlands; Janeczko et al. (36) considered both the urban environments (apartment and green suburbs) and two forests (coniferous and deciduous). For the cases of multiple exposures to the same environment, data were extracted immediately before the first exposure, and immediately after the final exposure.

Only data from the time points closest to the start and end times of the exposure were extracted, follow-up measures, or measurements taken during exposure, were not included in the analysis. The results were presented as the forest plots with 95% *CI*s. The *I*^2^-statistic was calculated to quantify the degree of heterogeneity between the studies (*I*^2^ = 0–25% represents no heterogeneity; *I*^2^ = 25–50% represents moderate heterogeneity; *I*^2^ = 50–75% represents large heterogeneity; and *I*^2^ = 75–100% represents extreme heterogeneity) (37). A random-effects model was employed for all the meta-analyses as it is considered to represent a more conservative approach, suitable for the cases of high heterogeneity (38). A sensitivity analysis was then performed. Additionally, a univariate meta-regression and a Galbraith radial plot were performed to explore the source of heterogeneity (38, 39).

Publication bias across the studies within the meta-analysis was first tested with the funnel plots using standardized mean difference (SMD) as the measure of study size on the vertical axis and mean difference on the horizontal axis. A symmetrical, inverted funnel indicates the absence of bias. In addition, a funnel plot asymmetry was tested using *Begg's* and *Egger's* tests (40), which examined the association between the effect estimates and their variances.

Quality of evidence was assessed using the Grading of Recommendations Assessment, Development, and Evaluation (GRADE) Working Group guideline (41, 42). The risk of bias, inconsistency, indirectness, imprecision, and publication bias are considered. The GRADE system classified the quality of evidence in one of four grades finally as “high,” “moderate,” “low,” and “very low.”

### Subgroup Analyses

The subgroup analyses were conducted based on the study characteristics (study region and study design), participant characteristics (mean age, sample size, and gender), and process of exposure (type of natural environment, type of built environment, and active category). Missingness was very limited. Two studies did not show the gender of the participants (35, 36). To accommodate the different age group categories across the included studies, we stratified pooled estimates for different age groups as those aged <45 and ≥45 years. The sample size was also grouped by the median (42).

All the statistical analyses were performed using the “metan” command in the STATA package version 15.1 software program, and a *p*-value <0.05 was defined as statistically significant.

## Results

**Figure 1:**
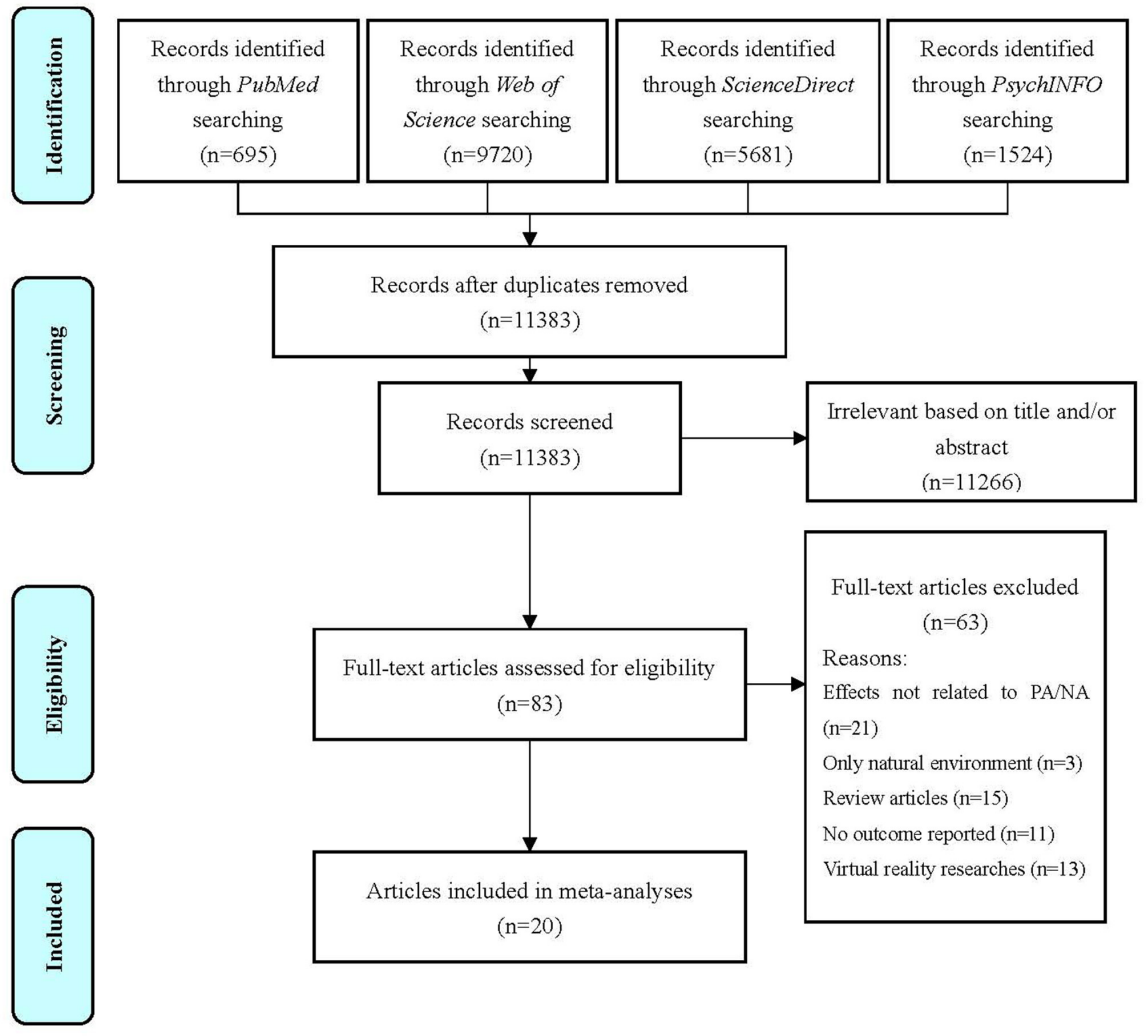
Flow diagram.

The initial database search yielded 17,620 articles, of which 6,237 were removed as repeats, and 11,266 were removed as clearly irrelevant after reviewing the titles and/or abstracts. We retrieved the full text of the 83 relevant papers. A further three studies were retrieved from the reference lists of the review articles. After independent assessment by the reviewer (WY), 20 articles met the inclusion criteria and were eligible for inclusion in the synthesis. A review flowchart is shown in Figure 1. The characteristics and synthesized results for all the 20 studies are presented in Table 1.

**Table 1:** Study characteristics.

**No**.	**Reference**	**Location**	**Study design**	**Sample**	**Natural environment exposure**	**Built environment exposure**	**Exposure procedure**	**Effect measures**
1	Berman et al. (16)	Canada (North America)	Mixed factorial design	*N* = 20 (12 females) Mean age: 26 Individuals diagnosed with MDD	Ann Arbor Arboretum	Traffic-heavy streets	50–55 min walking	PANAS
2	Bielinis et al. (43)	Poland (Europe)	Mixed factorial design	*N* = 62 (26 females) Mean age: 21.45 SD: 0.18 University students	Deciduous, broad-leaved forest	One of the most urbanized places in the city-street	15 min viewing	PANAS
3	Bielinis et al. (44)	Poland (Europe)	Between-subject	*N* = 32 (32 females) Mean age: 20.97 SD: 0.65 University students	Forest	Urban street	15 min viewing	PANAS
4	Bratman et al. (17)	United States (North America)	Mixed factorial design	*N* = 60 (33 females) Mean age: 22.9 Healthy adults	A park near Stanford University	A busy street	50 min walking	PANAS
5	Brooks et al. (45) (study 1)	Canada (North America)	Mixed factorial design	*N* = 121 (99 females) Mean age: 21.54 SD: 6.17 University students	Urban park	Hallways with few windows in the building	10 min walking	PANAS
6	Browning et al. (46)	United States (North America)	Between-subject	*N* = 82 (38 females) Mean age: 20.2 SD: 1.4 University students	59-acre bottomland oak-hickory forest	In front of a blank white wall.	6 min sitting + 6 min walking	PANAS
7	Calogiuri et al. (47)	Norway (Europe)	Between-subject	*N* = 14 (7 males) Mean age: 49 SD: 8 Employees in two workplaces	Outdoors in a park	A “typical” exercise setting (gym-hall)	25 min biking + 20 min strength session	PAAS
8	De Brito et al. (18)	United States (North America)	Within-subject	*N* = 23 (19 females) Mean age: 49.7 SD: 6.5 Healthy middle-aged adults	Minnesota landscape arboretum	Paved sidewalks adjacent to medium traffic roads	Once-weekly 50 min walking for 9 weeks	PANAS
9	Fuegen and Breitenbecher (48)	United States (North America)	Mixed factorial design	*N* = 180 (107 females) Mean age: 21.59 SD: 7.69 University students	University campus-natural	University campus-building	15 min viewing/walking	PANAS
10	Grazuleviciene et al. (49)	Lithuania (Europe)	Between-subject	*N* = 20 (7 females) Mean age: 62.3 SD: 12.6 Stable CAD patients	A beautiful pine park	A busy street	7 days exposure	PANAS
11	Hartig et al. (50) (study 2)	United States (North America)	Between-subject	*N* = 34 (17 males) Mean age: 20 University students	A park with a stream and associated riparian habitat	A well-kept area with mixed residential and commercial uses	40 min walking	ZIPERS
12	Janeczko et al. (36)	Poland (Europe)	Mixed factorial design	*N* = 75 Aged 19–24 University students	Multiple natural environment: coniferous forest; deciduous forest	Multiple built environment: apartment green suburbs	30 min walking	PANAS
13	Mayer et al. (51) (study 1)	United States (North America)	Mixed factorial design	*N* = 76 (51 females) University students	Nature preserves	A concrete area near a building with an adjacent parking lot	10 min walking	PANAS
14	Neill et al. (52) (study 1)	Canada (North America)	Mixed factorial design	*N* = 123 (104 females) Mean age: 21.02 SD: 3.7 University students	Urban park	Windowless laboratory room	5 min viewing	PANAS
15	Nisbet and Zelenski (53) (study 1)	Canada (North America)	Mixed factorial design	*N* = 150 (85 females) Mean age: 20.80 SD: 5.03 University students	Green corridor-university campus	Athletics building	17 min walking	PANAS
16	Olafsdottir et al. (54)	Luxembourg (Europe)	Between-subject	*N* = 67 (46 females) Mean age: 24.39 SD: 2.61 University students	A conserved and by far the largest recreational area-woodland.	Walking (on a treadmill) in a gym	40 min walking	PANAS
17	Reeves et al. (55)	England (Europe)	Mixed factorial design	*N* = 34 (21 females) Mean age: 41 SD: 10.28 The majority were contacted through a social corporate responsibility scheme	A managed “natural” wetland area	Urban bench outside the Center	430 m walking + 10 min viewing	PANAS
18	Takayama et al. (56)	Japan (Asia)	Mixed factorial design	*N* = 45 (0 females) Mean age: 21.13 Male university students	Four forest environments	Four urban environments: Along the downtown major traffic roads or around the main station	15 min walking + 15 min viewing	PANAS
19	Takayama et al. (57)	Japan (Asia)	Mixed factorial design	*N* = 46 (0 females) Mean age: 21.13 Male university students	Four forest environments	Four urban environments	15 min walking + 15 min viewing	PANAS
20	Tyrväinen et al. (35)	Finland (Europe)	Within-subject	*N* = 77 Mean age: 47.64 SD: 8.68 Participants work in the Helsinki Metropolitan Area	Multiple natural environment: urban park woodland	City center	15 min viewing + 30 min walking	PANAS

### Study Characteristics

Although there was no date restriction on the search, 90% of the studies were published in the past 10 years. The studies were conducted in nine different countries. Most were conducted in North America (*n* = 10), followed by Europe (*n* = 8), and Asia (*n* = 2).

#### Participants

The populations under investigation varied greatly in size, ranging from 14 (47) to 180 (48). Overall, nine studies included fewer than 50 subjects. The participants were typically young, with 13 studies recruiting the college or university students. A significant difference was found in the sample populations, which included the university students, healthy adults (17, 18), full-time regularly employed people (35, 47), and subjects with specific mental or physical illnesses, such as major depressive disorder (16), and coronary artery disease (49).

#### Outcome Measures

We recorded the instruments used to assess current emotional state, which included the Positive and Negative Affective Schedule (PANAS, *n* = 18), the Zuckerman Inventory of Personal Reactions (ZIPERS, *n* = 1), and the Physical Activity Affective Scale (PAAS, *n* = 1). All the 20 studies assessed the positive affect changes and 18 studies assessed the negative affect changes after exposure to the natural environment, two studies reported on positive affect only (47, 50).

#### Intervention

The experimental time ranged from 5 (52) to 55 min (16) within 1 day. Two studies had a considerably longer exposure time whereby they completed their experiment in 7 days (49) and 9 weeks (18). For the exposure procedure, 10 studies had participants walking in the environment for a period of time, and the three studies (43, 44, 52) asked participants to complete a viewing. Six studies had multiple exposure (walking and/or viewing) methods (35, 46, 48, 55–57). One study (47) had participants exercise (25 min biking + 20 min strength session) in a green/natural area or in an indoor exercise setting.

Most of the studies used forests (*n* = 7) or urban parks (*n* = 6) as their natural environment. Four studies used the natural environments characterized by their biodiversity, which included the arboretum (16, 18), wetland (55), and natural preserves (51). Two studies used the university campuses (48, 53). One study used multiple natural environments (woodland and urban parks) (35). For a comparative built environment, most of the studies described an urbanized location on a city street (*n* = 8) and building site (*n* = 7). Three studies used the indoor environments (hallways, blank white walls, and gym-halls). One used a parking lot (51) and one used an urban bench outside the center (55).

### Study Quality

All the 20 studies were assessed for quality using an adapted version of the Twohig-Bennett and Jones (30) and Ogilvie et al. (31) risk of bias tool. The quality assessments were initially done by the first reviewer (WY) and then all the studies were cross-checked by another (FC) for discrepancies.

For the 20 studies detailed in Supplementary Table 4, the scores ranged from 6 to 10 out of a total of 10 criteria. One study scored 10 (16), and 80% of the studies scored ≥8. Three studies scored 7 (45, 50, 53) and one scored 6 (48). The two criteria that were the most recurrently missing from the studies were “*4. Exposure: Did the authors show that participants did not receive concurrent intervention which could have influenced the results (no explanation scores zero)?*” and “*5. Representativeness: Were the study samples shown to be representative of the study population?*”

### Meta-Analysis

**Figure 2:**
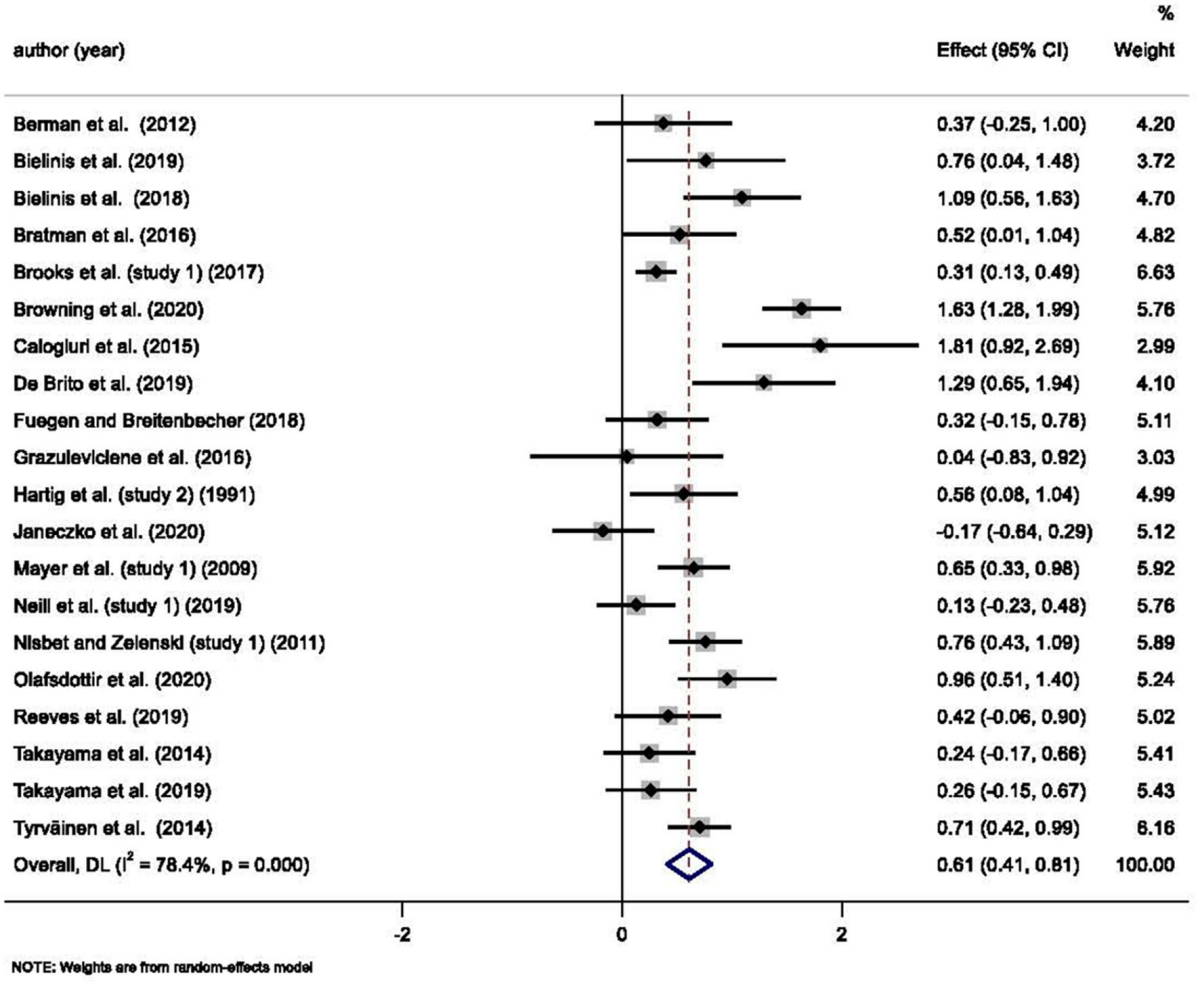
Meta-analysis results for the association between natural and built environment exposure on positive affect.

**Figure 3:**
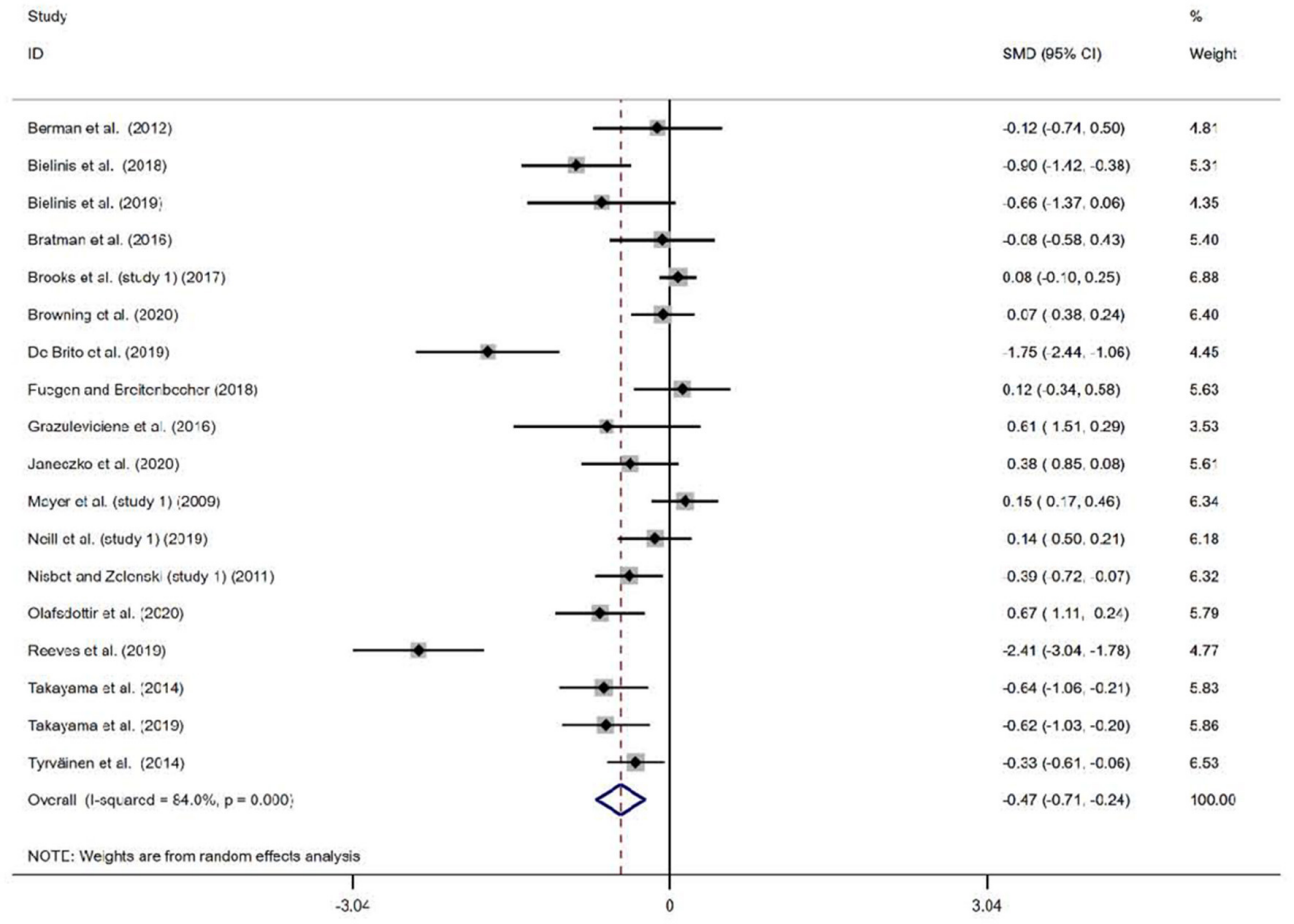
Meta-analysis results for the association between natural and built environment exposure on negative affect.

The results of the 20 studies and the meta-analysis estimates are shown in Figures 2, 3. Most of the studies reported that the natural environment was associated with a significant increase in positive affect (SMD = 0.61, 95% *CI* 0.41, 0.81, *p* < 0.001) and decreased negative affect (SMD = −0.47, 95% *CI* −0.71, −0.24, *p* < 0.001). The observed positive affect sizes ranged from −0.17 to 1.81, and the negative affect sizes ranged from −2.41 to 0.15. The *I*^2^ scores for both the positive and negative affect meta-analyses were higher than 75%, indicating extreme heterogeneity between the studies. To test whether significant the meta-analysis results were due to the inclusion of poor-quality studies, a sensitivity analysis was conducted where possible. The sensitivity analyses excluding single studies did not materially change the pooled results. Supplementary Figures 1, 2 show the results from the sensitivity analysis.

A univariate meta-regression was performed to explore the source of heterogeneity for the meta-analysis with ≥10 studies included (38). The variables included study region, study design, sample size, mean age, percentage of females, total exposure time, type of natural environment, type of built environment, and active category (Supplementary Tables 5, 6). The results showed that a study design was associated with positive affect differences. The study design contributed to the heterogeneity with *tau*^2^ reduced from 0.1534 to 0.0940. The mean age (coefficient = −0.0246, *p* = 0.040) and sample size (coefficient = 0.0072, *p* = 0.011) were significantly associated with negative affect. The other variables did not reach significance. The results from the Galbraith plot (Supplementary Figures 3, 4) indicated that the studies with the highest positive affect (46) and lowest positive affect (55) may have been the main cause of the high heterogeneity. After excluding those two studies, the adjusted positive affect was 0.54 (95% *CI* 0.37–0.70, *I*^2^ = 63.9%, *p* < 0.001), and approximately 14.5% of the heterogeneity was attributed to the study of Browning et al. (46). The negative affect was −0.36 (95% *CI* −0.54 to −0.17, *I*^2^ = 73.5%, *p* < 0.001), and 10.5% of the heterogeneity was attributed to the study by Reeves et al. (55). Additionally, our confidence in the cumulative evidence was “low” for natural exposure to positive affect, and was “very low” for negative affect according to GRADE system (Supplementary Table 7).

**Figure 4:**
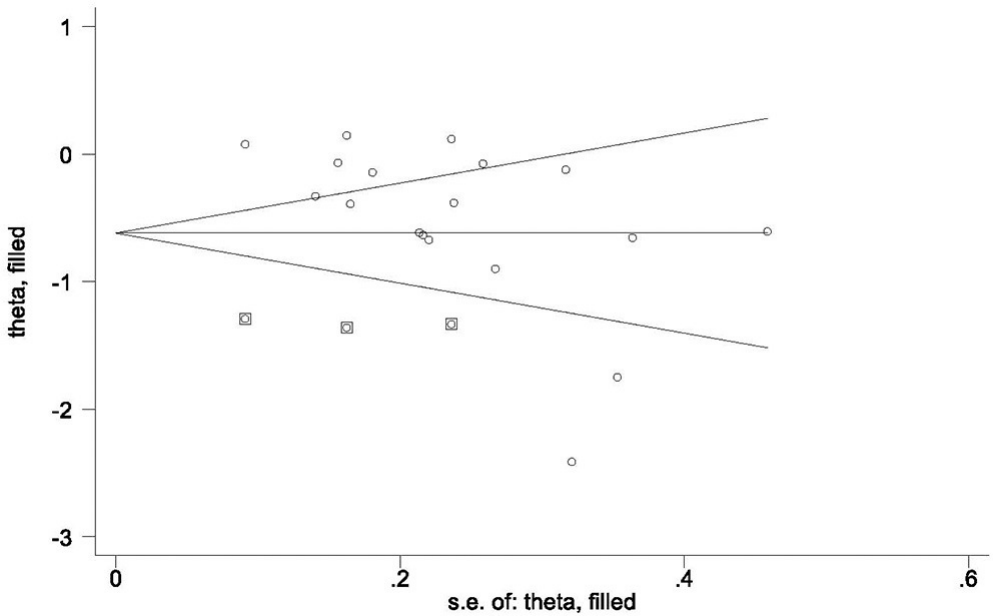
Filled funnel plot to access potential publication bias of the negative affect studies (*n* = 18). The circle and square with circle inside represent observed data (18 comparisons) and data added (three studies) by the trim-and-fill analysis.

Using the funnel plots (Supplementary Figures 5, 6), the studies of the positive affect were identified as visually symmetrical, both *Egger's* (*P*_*POS*_ = 0.217) and *Begg's* (*P*_*POS*_ = 0.206) tests of funnel asymmetry were non-significant, suggesting that the effects of publication bias on positive affect were negligible. However, both *Egger's* and *Begg's* (*P*_*NEG*_ = 0.019) tests indicated the presence of publication bias on negative affect. The trim and fill analysis suggested that the three studies were missing from our dataset (Figure 4). After adding the missing data to the original dataset, the reported significant effects of the natural environment on the negative affect were intact (SMD = −0.62, 95% *CI* −0.90, −0.34, *p* < 0.001), suggesting that the effects of publication bias on the negative affect were negligible.

### Subgroup Analyses

The subgroup analyses of the study characteristics, the participant characteristics and process of exposure identified the effect of the natural environment on positive and negative affect. A summary of the subgroup analysis is given in Tables 2, 3. As shown in Table 2, the older samples (effect size = 0.941) exposed to the natural environment were found to be more positive. Furthermore, we also assessed whether the effect size differed based on the type of natural and built environment, and found that biodiverse areas (effect size = 0.654) and forests (effect size = 0.682) had a higher effect on positive emotion. Moreover, the natural environment had a stronger effect on the positive emotions than the indoor environment (effect size = 1.208).

**Table 2:** Summary of subgroup analysis of positive affect studies (*n* = 20) conducted by study characteristics, participants characteristics, and process of exposure.

**Subgroups**	***N***	**Effect**	**LCI**	**UCI**	***I^**2**^***	***p*-value**	**Heterogeneity between groups**
**Study characteristics**							
Study region							*p* = 0.103
North America	10	0.647	0.347	0.946	84.1%	<0.001	
Europe	8	0.670	0.313	1.028	73.2%	<0.001	
Asia	2	0.253	−0.039	0.544	0%	0.957	
Study design							***p*** **=** **0.027**
Within-subject	2	0.923	0.369	1.477	62.4%	0.103	
Between-subject	6	0.980	0.486	1.474	78.0%	<0.001	
Mixed factorial design	12	0.401	0.235	0.567	53.5%	0.014	
**Participants characteristics**							
Mean age							*p* = 0.219
18–45	16	0.549	0.328	0.769	79.2%	<0.001	
≥45	4	0.941	0.355	1.526	71.2%	0.015	
Sample size							*p* = 0.721
≥median number of sample	10	0.635	0.338	0.933	86.9%	<0.001	
< median number of sample	10	0.563	0.305	0.822	51.9%	0.028	
Gender							*p* = 0.095
Male	2	0.253	−0.039	0.544	0%	0.957	
Female	1	0.762	0.042	1.481	–	–	
Mixed	15	0.614	0.434	0.795	63.2%	0.001	
**Process of exposure**							
Type of natural environment							*p* = 0.801
Biodiverse area	4	0.654	0.319	0.989	44.8%	0.142	
Forest	7	0.682	0.171	1.193	88.6%	<0.001	
Urban park	6	0.452	0.147	0.758	63.3%	0.018	
University campus	2	0.569	0.140	0.999	56.6%	0.129	
Type of built environment							*p* = 0.628
City-street	8	0.565	0.275	0.855	52.9%	0.038	
Building site	7	0.475	0.196	0.755	72.1%	0.001	
Indoor	3	1.208	0.125	2.292	96.0%	<0.001	
Active category							*p* = 0.997
Active	11	0.601	0.352	0.851	71.4%	<0.001	
Passive	3	0.629	−0.025	1.283	78.8%	0.009	
Mixed	6	0.607	0.159	1.056	87.4%	<0.001	

*N, Number of study group; LCI, Lower confidence interval; UCI, Upper Confidence Intervals*.

**Table 3:** Summary of subgroup analysis of negative affect studies (*n* = 18) conducted by study characteristics, participants characteristics, and process of exposure.

**Subgroups**	***N***	**Effect**	**LCI**	***UCI***	***I* ^**2**^**	***p*-value**	**Heterogeneity between groups**
**Study characteristics**							
Study region							***p*** **=** **0.016**
North America	9	−0.173	−0.415	0.069	74.7%	<0.001	
Europe	7	−0.833	−1.310	−0.357	84.0%	<0.001	
Asia	2	−0.626	−0.924	−0.328	0%	0.948	
Study design							*p* = 0.889
Within-subject	2	−1.003	−2.392	0.385	92.8%	<0.001	
Between-subject	4	−0.433	−0.816	−0.050	53.2%	0.094	
Mixed factorial design	12	−0.410	−0.712	−0.108	86.3%	<0.001	
**Participants characteristics**							
Mean age							*p* = 0.343
18–45	15	−0.411	−0.664	−0.158	83.9%	<0.001	
≥45	3	−0.873	−1.795	0.049	85.7%	0.001	
Sample size							***p*** **=** **0.006**
≥median number of sample	9	−0.164	−0.339	0.011	61.9%	0.007	
< median number of sample	9	−0.853	−1.312	−0.394	82.9%	<0.001	
Gender							*p* = 0.805
Male	2	−0.626	−0.924	−0.328	0%	0.948	
Female	1	−0.657	−1.369	−0.056	-	-	
Mixed	13	−0.492	−0.804	−0.180	87.6%	<0.001	
**Process of exposure**							
Type of natural environment							***p*** **=** **0.003**
Biodiverse area	4	−1.019	−2.279	0.241	95.4%	<0.001	
Forest	7	−0.523	−0.757	−0.289	47.9%	0.074	
Urban park	4	−0.006	−0.170	0.158	5.6%	0.365	
University campus	2	−0.165	−0.662	0.333	68.2%	0.076	
Type of built environment							***p*** **<** **0.001**
City-street	8	−0.650	−0.974	−0.325	61.7%	0.011	
Building site	6	−0.308	−0.490	−0.125	30.9%	0.204	
Indoor	2	0.040	−0.115	0.194	0%	0.421	
Active category							*p* = 0.652
Active	9	−0.356	−0.662	−0.050	80.0%	<0.001	
Passive	3	−0.528	−1.044	−0.013	66.7%	<0.001	
Mixed	6	−0.616	−1.126	−0.105	90.2%	<0.001	

*N, Number of study group; LCI, Lower confidence interval; UCI, Upper Confidence Intervals*.

For the effect on negative affect, the results indicated a significant difference between the study region (*p* = 0.016), sample size (*p* = 0.006), type of natural (*p* = 0.003), and built (*p* < 0.001) environments. A larger effect size was observed in the studies conducted in Europe (effect size = −0.833). The older samples (effect size = −0.873) and the smaller samples (effect size = −0.853) were found to be more effective during exposure to the natural environment. A subgroup analysis based on the process of exposure revealed that exposure of the participants to the biodiverse areas was associated with a significant reduction in negative affect (effect size = −1.019).

## Discussion

This systematic review and meta-analysis of 20 studies from 9 countries provided evidence that exposure to the natural environment is associated with the positive and negative affect. The meta-analysis results have showed that brief contact of the participants with the natural environment was associated with the higher levels of positive affect and the lower levels of negative affect.

This analysis is similar to the results of a previous meta-analysis which found that the type of emotion assessment, type of exposure to nature, study location, and mean age of the participants significantly moderated the effect of the natural environment on positive and negative mood (58). In that meta-analysis, the authors assessed the mean effect size of exposure to the natural environments on both positive and negative affect. They included 32 studies from 23 articles that used a randomized controlled design. By comparison, we included all the empirical studies that focused on both the natural and built environments associated with positive and negative affect. This provided a more precise estimate of the difference between the effects of the natural and built environments on positive and negative affect. In addition, the prior meta-analysis was published in 2015, and only included studies published before 2010. In our analysis, 90% of the studies published between 2010 and 2020, highlighted recent developments in the natural environment and psychological health. In addition to study quality, which is different with the previous research, we had performed a risk of bias assessment for each study, a sensitivity analysis, a univariate meta-regression, and a Galbraith radial plot to explore the sources of heterogeneity, and the funnel plots were made to assess the potential publication bias, GRADE system was also used to estimates the quality of evidence. Collectively, our systematic review and meta-analysis builds on the prior meta-analysis, strengthening the contrast between the natural and built environments, and performing more thorough assessments of the included studies. The evidence from our study may be more comprehensive and precise. Our findings are consistent with those of a previous study from some perspectives. The results showed that exposure to the natural environment was associated with an increase in positive affect (SMD = 0.61, 95% *CI* 0.41, 0.81) and a decrease in negative affect (SMD = −0.47, 95% *CI* −0.71, −0.24) relative to the comparison conditions, which is consistent with a prior study (positive affect: effect size estimate = 0.31, 95% *CI* 0.24, 0.37; negative affect, effect size estimate = −0.12, 95% *CI* −0.17, −0.07). Our findings also converge with previous research that demonstrated that exposure to nature is beneficial to health (10, 30).

The results showed extreme heterogeneity ($I_{POS}^{2}$ = 78.4%, $I_{NEG}^{2}$ = 84.0%) between the studies, which remained largely unexplained following a sensitivity analysis. We used the meta-regression models to probe the source of heterogeneity. The univariate meta-regression results showed that study design, study region, mean age, and sample size contributed to the heterogeneity. The model showed that study design was associated with positive affect between natural and built environment. Study region, mean age (*p* = 0.040) and sample size (*p* = 0.011) were related to negative affect between natural and built environment. Other variables did not reach the significance level (Supplementary Tables 5, 6). A Galbraith plot indicated that the two studies (46, 55) may have been the main cause of the high heterogeneity. After excluding these two studies, the *I*^2^*-*values decreased ($I_{POS}^{2}$ = 63.9%, $I_{NEG}^{2}$ =73.5%). However, the meta-analysis results still showed large heterogeneity. Due to high between-study heterogeneity, the confidence in the pooled estimates were graded as “low” or “very low.” Therefore, the future meta-analyses should include more scientific empirical studies to draw more definitive conclusions.

During the subgroup analysis, we examined factors that may moderate the effect of nature on the positive and negative emotions. The results of these analyses indicated that the study region, study design, mean age of the sample, sample size, and type of natural and built environment were all important factors contributing to increased positive affect and decreased negative affect. As shown in Table 2, the positive effect was 0.923 (95% *CI* 0.369–1.477), 0.980 (95% *CI* 0.486–1.474), and 0.401 (95% *CI* 0.235–0.567), respectively. The study design with within-subject and between-subject got the higher positive affect. This finding was a supplement to the results of previous reports (10, 58). However, significant heterogeneity was found between the groups (*p* = 0.027), which was consistent with the results of the meta-regression models. On the other hand, study region, sample size, type of natural, and built environment were all found significantly heterogeneity between the groups in the subgroup analysis of negative affect, this might be explained by the potential bias in the included studies and “low” or “very low” quality evidence. To identify the participant characteristic changes in positive and negative affect between natural and built environment, mean age, sample size and gender were considered and the studies were divided into 2–3 groups, i.e., the studies were divided into two groups (18–45, ≥45) according to mean age of the participants. The older samples exposed to natural environment were found to have more positive (effect size = 0.941) and fewer negative (effect size = −0.873) emotions than those exposed to a built environment. This finding was consistent with a previous study (58) and should be taken seriously. There are 16 studies recruiting college students or youth groups in our pooled studies. Future research should pay more attention to the elderly. In addition, a smaller sample size (effect size = −0.853) was found to be significantly associated with a reduction in the negative emotions, which might be explained by the different experimental procedures. Nisbet and Zelenski (53) made all the participants (indoor walking route, *n* = 78; outdoor walking route, *n* = 72) walk along their assigned routes. Interference between the participants was inevitable. Another subgroup meta-analysis was based on the process of exposure. The positive affect was 0.654 (95% *CI* 0.319–0.989), 0.682 (95% *CI* 0.171–1.193), 0.452 (95% *CI* 0.147–0.758), and 0.569 (95% *CI* 0.140–0.999) for biodiverse area, forest, urban park, and university campus, respectively. Thus, the discrepancy was stable regardless of the type of natural environment for positive affect, suggesting that contact with managed nature and wild nature produces similar effect for positive emotion. In contrast, the negative affect was −1.019 (95% *CI* −2.279 to 0.241), −0.523 (95% *CI* −0.757 to −0.289), −0.006 (95% CI −0.170 to 0.158), and −0.165 (95% CI −0.662 to 0.333) for biodiverse area, forest, urban park, and university campus, respectively. The findings indicated significant moderation, with exposure to wild natural environments having a greater effect on decreasing negative affect than exposure to the managed natural environments. As a result, managed nature could serve as substitute for wild nature to increase positive emotions, while this is not suitable for alleviating negative emotions.

## Strengths and Limitations

The current study provides the most comprehensive and up-to-date findings on the effect of exposure to the natural environment on positive and negative affect. Its key strengths are its broad range of included studies, and its fully reproducible and transparent meta-analysis approach. Second, we assessed the quality and risk of bias for each included study according to the validated scales and determined confidence of our pooled estimates according to the GRADE system. Thus, our results may be valuable for the researchers in this area and improve quality of future research. Third, we conducted the subgroup analyses to show the positive and negative affect between natural and built environment, potential moderators of the effect of natural on positive and negative affect were discussed, thus, to help clarify the manner in which nature contributes to human well-being.

However, our study had some limitations. First, there was extreme heterogeneity in all of the pooled studies. Second, according to the GRADE system, the credibility of the cumulative evidence was “low” or “very low.” The main causes may include high heterogeneity, and inconsistent results across the studies. Third, the included studies were not representative of global populations, as half were from North America and, the others were form Europe and Asia, so our results may not be generalizable to the other areas. Fourth, several included studies used the small samples, which tend to provide a less stable estimate of the effect size. Fifth, nearly all our included studies controlled for potential confounders, yet confounding factors varied among the studies, and some important confounding factors, such as the quantity of greenspace were not considered.

## Conclusion

In summary, the present systematic review and meta-analysis indicated an association between the natural environment and positive and negative affect. However, owing to extreme heterogeneity, it is difficult to draw a robust conclusion. Future research in this area should take appropriate steps to reduce bias and improve quality to build a stronger evidence-based medical foundation. The research focus should also be extended to other geographic areas. The advanced methods should be applied to assess the positive and negative effects. Further technological refinement is required to become a viable tool to support evidence-based decision-making for public health and greenspace provision. Some important confounding variables, such as meteorological factors and, quantity and quality of greenspace should be collected and adjusted. Finally, although our systematic review uncovered a large body of research on the relationship between the environment and positive and negative affect, there is a paucity of studies on the mechanisms underlying this relationship. The mechanistic studies are needed to clarify the manner in which nature contributes to the development of human feelings and functioning, which could possibly answer more questions regarding the nature of the relationship between human health and the environment.

## Data Availability Statement

The original contributions presented in the study are included in the article/Supplementary Material, further inquiries can be directed to the corresponding author.

## Author Contributions

WY: conceptualization, project administration, funding acquisition, and writing. WY, SW, and XZ: assessment and analysis. WY and FC: data retrieval, curation, and methodology. All authors made substantial contributions to the study. All authors contributed to the article and approved the submitted version.

## Funding

This work was funded by the National Natural Science Foundation of China (No.32001367) and Open Fund of innovation institute for Sustainable Maritime Architecture Research and Technology (iSMART), Qingdao University of Technology (No. C2020-037).

## Conflict of Interest

The authors declare that the research was conducted in the absence of any commercial or financial relationships that could be construed as a potential conflict of interest.

## Publisher's Note

All claims expressed in this article are solely those of the authors and do not necessarily represent those of their affiliated organizations, or those of the publisher, the editors and the reviewers. Any product that may be evaluated in this article, or claim that may be made by its manufacturer, is not guaranteed or endorsed by the publisher.
