# Supplementary material for: Impact of Exposure to Natural and Built Environments on Positive and Negative Affect: A Systematic Review and Meta-Analysis
Source: Front Public Health. 2021 Nov 25;9:758457. doi: 10.3389/fpubh.2021.758457 (PMC8655776; doi:10.3389/fpubh.2021.758457)
Supplement: Supplementary file 1 [file Data_Sheet_1.docx]

**SUPPLEMENTARY MATERIALS**

**Tab. S1.** **PRISMA checklist.**

| **Section/topic** | **#** | **Checklist item** | **Reported on page #** |
| --- | --- | --- | --- |
| **TITLE** | | |  |
| Title | 1 | Identify the report as a systematic review, meta-analysis, or both. | 1 |
| **ABSTRACT** | | |  |
| Structured summary | 2 | Provide a structured summary including, as applicable: background; objectives; data sources; study eligibility criteria, participants, and interventions; study appraisal and synthesis methods; results; limitations; conclusions and implications of key findings; systematic review registration number. | 1 |
| **INTRODUCTION** | | |  |
| Rationale | 3 | Describe the rationale for the review in the context of what is already known. | 2 |
| Objectives | 4 | Provide an explicit statement of questions being addressed with reference to participants, interventions, comparisons, outcomes, and study design (PICOS). | 2-3 |
| **METHODS** | | |  |
| Protocol and registration | 5 | Indicate if a review protocol exists, if and where it can be accessed (e.g., Web address), and, if available, provide registration information including registration number. | 3 |
| Eligibility criteria | 6 | Specify study characteristics (e.g., PICOS, length of follow-up) and report characteristics (e.g., years considered, language, publication status) used as criteria for eligibility, giving rationale. | 3-4 |
| Information sources | 7 | Describe all information sources (e.g., databases with dates of coverage, contact with study authors to identify additional studies) in the search and date last searched. | 3 |
| Search | 8 | Present full electronic search strategy for at least one database, including any limits used, such that it could be repeated. | 4 |
| Study selection | 9 | State the process for selecting studies (i.e., screening, eligibility, included in systematic review, and, if applicable, included in the meta-analysis). | 4 |
| Data collection process | 10 | Describe method of data extraction from reports (e.g., piloted forms, independently, in duplicate) and any processes for obtaining and confirming data from investigators. | 4 |
| Data items | 11 | List and define all variables for which data were sought (e.g., PICOS, funding sources) and any assumptions and simplifications made. | 3-4 |
| Risk of bias in individual studies | 12 | Describe methods used for assessing risk of bias of individual studies (including specification of whether this was done at the study or outcome level), and how this information is to be used in any data synthesis. | 4 |
| Summary measures | 13 | State the principal summary measures (e.g., risk ratio, difference in means). | 4-5 |
| **Section/topic** | **#** | **Checklist item** | **Reported on page #** |
| Synthesis of results | 14 | Describe the methods of handling data and combining results of studies, if done, including measures of consistency (e.g., I^2^) for each meta-analysis. | 5-6 |
| Risk of bias across studies | 15 | Specify any assessment of risk of bias that may affect the cumulative evidence (e.g., publication bias, selective reporting within studies). | 5 |
| Additional analyses | 16 | Describe methods of additional analyses (e.g., sensitivity or subgroup analyses, meta-regression), if done, indicating which were pre-specified. | 5-7 |
| **RESULTS** | | |  |
| Study selection | 17 | Give numbers of studies screened, assessed for eligibility, and included in the review, with reasons for exclusions at each stage, ideally with a flow diagram. | 6 |
| Study characteristics | 18 | For each study, present characteristics for which data were extracted (e.g., study size, PICOS, follow-up period) and provide the citations. | 6 |
| Risk of bias within studies | 19 | Present data on risk of bias of each study and, if available, any outcome level assessment (see item 12). | 6 |
| Results of individual studies | 20 | For all outcomes considered (benefits or harms), present, for each study: (a) simple summary data for each intervention group (b) effect estimates and confidence intervals, ideally with a forest plot. | Supp Material |
| Synthesis of results | 21 | Present results of each meta-analysis done, including confidence intervals and measures of consistency. | 6-7 |
| Risk of bias across studies | 22 | Present results of any assessment of risk of bias across studies (see Item 15). | 7 |
| Additional analysis | 23 | Give results of additional analyses, if done (e.g., sensitivity or subgroup analyses, meta-regression [see Item 16]). | 8-9 |
| **DISCUSSION** | | |  |
| Summary of evidence | 24 | Summarize the main findings including the strength of evidence for each main outcome; consider their relevance to key groups (e.g., healthcare providers, users, and policy makers). | 9-10 |
| Limitations | 25 | Discuss limitations at study and outcome level (e.g., risk of bias), and at review-level (e.g., incomplete retrieval of identified research, reporting bias). | 10 |
| Conclusions | 26 | Provide a general interpretation of the results in the context of other evidence, and implications for future research. | 11 |
| **FUNDING** | | | |
| Funding | 27 | Describe sources of funding for the systematic review and other support (e.g., supply of data); role of funders for the systematic review. | 11 |

*From*: Moher D, Liberati A, Tetzlaff J, Altman DG, The PRISMA Group (2009). Preferred Reporting Items for Systematic Reviews and Meta-Analyses: The PRISMA Statement. PLoS Med 6(7): e1000097. doi:10.1371/journal. pmed1000097

**Tab. S2 Search strategies for all databases searched.**

| **Database** | **Search strategies** |
| --- | --- |
| ***Web of Science*** | 1. TS= (“natural environment*” OR “natural landscape” OR “exposure to nature” OR “natural space” OR “green space*” OR “greenspace” OR “urban forest” OR “green exercise” OR “greening” OR “greenness” OR park OR “gardening” OR nature OR “urban environment” OR “viewing landscape” OR “forest bathing” OR “forest walking” OR “forest environment” OR “benefi* of nature” OR "effect*of nature" OR “restorative environment” OR “urban green environments” OR “urban park” OR “urbanization” OR landscape OR “therapeutic landscape” OR “urban greenspace” OR “healing environment”)  2. TS= (“positive affect” OR “positive emotion” OR “negative affect” OR restoration OR “negative emotion” OR “well-being” OR “psychological benefits” OR “psychological traits” OR wellbeing OR psychological OR “emotion*” OR mood)  3 TS= (participants OR students OR subjects OR volunteers OR people)  4. 1 AND 2 AND 3 |
| ***PubMed*** | 1. (“natural environment” OR “exposure to nature” OR “natural space” OR “greenspace” OR “urban forest” OR “green exercise” OR “greening” OR “greenness” OR “urban environment” OR “viewing landscape” OR “forest bathing” OR “forest walking” OR “forest environment” OR “restorative environment” OR “urban green environments” OR “urban park” OR “healing environment”)  2. (“positive affect” OR “positive emotion” OR “negative affect” OR restoration OR “negative emotion” OR “well-being” OR “psychological benefits” OR “psychological traits” OR wellbeing OR “emotion*” OR mood)  3. (“control group” OR “non-randomized” OR “crossover procedure” OR “field experiment” OR “randomized controlled trial” OR randomized OR intervention)  4. animal/  5. 1 AND 2 AND 3  6. 5 NOT 4 |
| ***ScienceDirect*** | (“natural environment” OR “urban park” OR “urban environment” OR “therapeutic landscape”) AND (“positive affect” OR “positive emotion” OR “negative emotion” OR “negative affect”) |
| ***PsychINFO*** | (“negative affect” OR “negative emotion” OR “positive affect” OR “positive emotion” OR “affect” OR “affective well-being” OR “emotion” OR “emotional well-being” OR “happiness” OR “restoration” OR “wellbeing”) AND (“nature” OR “natural” OR “natural environments”) |

**Tab. S3: Adapted Hanson and Jones and Ogilvie et al. risk of bias tool for intervention studies.**

| **Item Methodological quality** | **Description** | **Scale** |
| --- | --- | --- |
| 1. Reporting: outcome(s) | Are the main outcomes to be measured clearly described in the introduction or methods section? (if the main outcomes are first mentioned in the results section, this question should be answered no) | 1: Yes – clearly described in introduction /methods  0: No – not clearly described/first mentioned in results |
| 2. Reporting: intervention | Are the interventions of interest (greenspace and control or otherwise) clearly described? | 1: Yes – clearly described  0: No |
| 3. Randomisation | Were participants, groups, or areas randomly allocated to intervention and control status (no randomised and no explanation scores zero)? | 1: Yes – description of a randomisation process  0: No – no explanation |
| 4. Exposure | Did the authors show that participants did not receive concurrent intervention which could have influenced the results (no explanation scores zero)? | 1: Yes  0: No – no explanation |
| 5. Representativeness | Were the study samples shown to be representative of the study population? | 1: Yes – shown to be representative  0: No – shown not to be representative  N: Insufficiently described |
| 6. Comparability | Were baseline characteristics of intervention and control groups, populations, or areas comparable, or if there were important differences in potential confounders at baseline were these appropriately adjusted for in analysis? | 1: Yes  0: No  N: Insufficiently described |
| 7. Attrition | Were numbers of participants at follow-up identifiable as at least 80% of the baseline? | 1: Yes  0: No |
| 8. Tools | Were valid and reliable tools used to assess participant outcomes? | 1: Yes  0: No |
| 9. Follow-up time scale | Was the length of time to follow up assessment appropriate for the intervention? | 1: Yes  0: No |
| 10. Precision of the results | Were confidence intervals or p-values given? | 1: Yes  0: No |

**Tab. S4: Quality appraisal results for intervention studies using an adapted version of the Twohig-Bennett and Jones and Ogilvie et al. risk of bias tool**.

|  | **Risk of bias checklist results** | | | | | | | | | | |
| --- | --- | --- | --- | --- | --- | --- | --- | --- | --- | --- | --- |
| **Lead author, year** | 1 | 2 | 3 | 4 | 5 | 6 | 7 | 8 | 9 | 10 | **Total score (out of 10)** |
| Berman et al., 2012 | 1 | 1 | 1 | 1 | 1 | 1 | 1 | 1 | 1 | 1 | 10 |
| Bielinis et al., 2019 | 1 | 1 | 1 | 0 | N | 1 | 1 | 1 | 1 | 1 | 8 |
| Bielinis et al., 2018 | 1 | 1 | 1 | 0 | N | 1 | 1 | 1 | 1 | 1 | 8 |
| Bratman et al. ,2015 | 1 | 1 | 1 | 1 | N | 1 | 1 | 1 | 1 | 1 | 9 |
| Brooks et al., 2017 (study 1) | 1 | 1 | 1 | 0 | N | N | 1 | 1 | 1 | 1 | 7 |
| Browning et al., 2020 | 1 | 1 | 1 | 1 | 1 | N | 1 | 1 | 1 | 1 | 9 |
| Calogiuri et al., 2015 | 1 | 1 | 1 | 0 | 1 | 1 | 1 | 1 | 1 | 1 | 9 |
| De Brito et al., 2019 | 1 | 1 | 0 | 1 | 1 | 1 | 1 | 1 | 1 | 1 | 9 |
| Fuegen and Breitenbecher, 2018 | 1 | 0 | 1 | 0 | N | 1 | 0 | 1 | 1 | 1 | 6 |
| Grazuleviciene et al., 2016 | 1 | 1 | 1 | 0 | N | 1 | 1 | 1 | 1 | 1 | 8 |
| Hartig et al., 1991 (study 2) | 1 | 1 | 1 | 0 | N | N | 1 | 1 | 1 | 1 | 7 |
| Janeczko et al., 2020 | 1 | 1 | 0 | 0 | 1 | 1 | 1 | 1 | 1 | 1 | 8 |
| Mayer and Frantz et al., 2009 (study 1) | 1 | 1 | 1 | 1 | N | N | 1 | 1 | 1 | 1 | 8 |
| Neill et al., 2019 (study 1) | 1 | 1 | 1 | 0 | N | 1 | 1 | 1 | 1 | 1 | 8 |
| Nisbet and Zelenski, 2011 (study 1) | 1 | 1 | 1 | 0 | N | N | 1 | 1 | 1 | 1 | 7 |
| Olafsdottir et al., 2020 | 1 | 1 | 1 | 0 | 1 | 1 | 0 | 1 | 1 | 1 | 8 |
| Reeves et al., 2019 | 1 | 1 | 0 | 0 | 1 | 1 | 1 | 1 | 1 | 1 | 8 |
| Takayama et al. 2014 | 1 | 1 | 1 | 0 | 1 | 1 | 1 | 1 | 1 | 1 | 9 |
| Takayama et al., 2019 | 1 | 1 | 1 | 0 | 1 | 1 | 1 | 1 | 1 | 1 | 9 |
| Tyrväinen et al., 2014 | 1 | 1 | 1 | 0 | N | 1 | 1 | 1 | 1 | 1 | 8 |


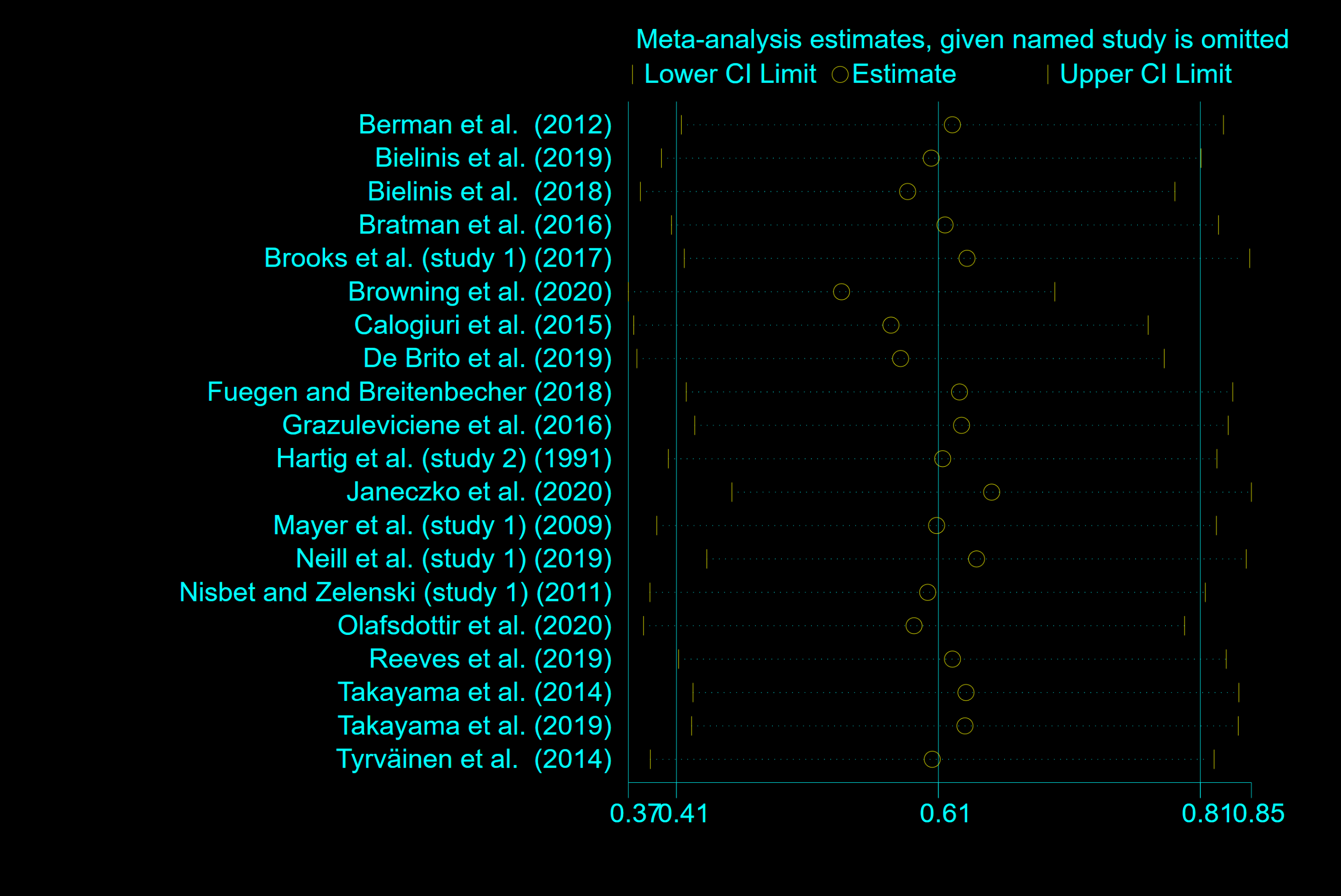


**Fig.S1 Sensitivity analysis of the association between** **natural exposure and positive affect.**


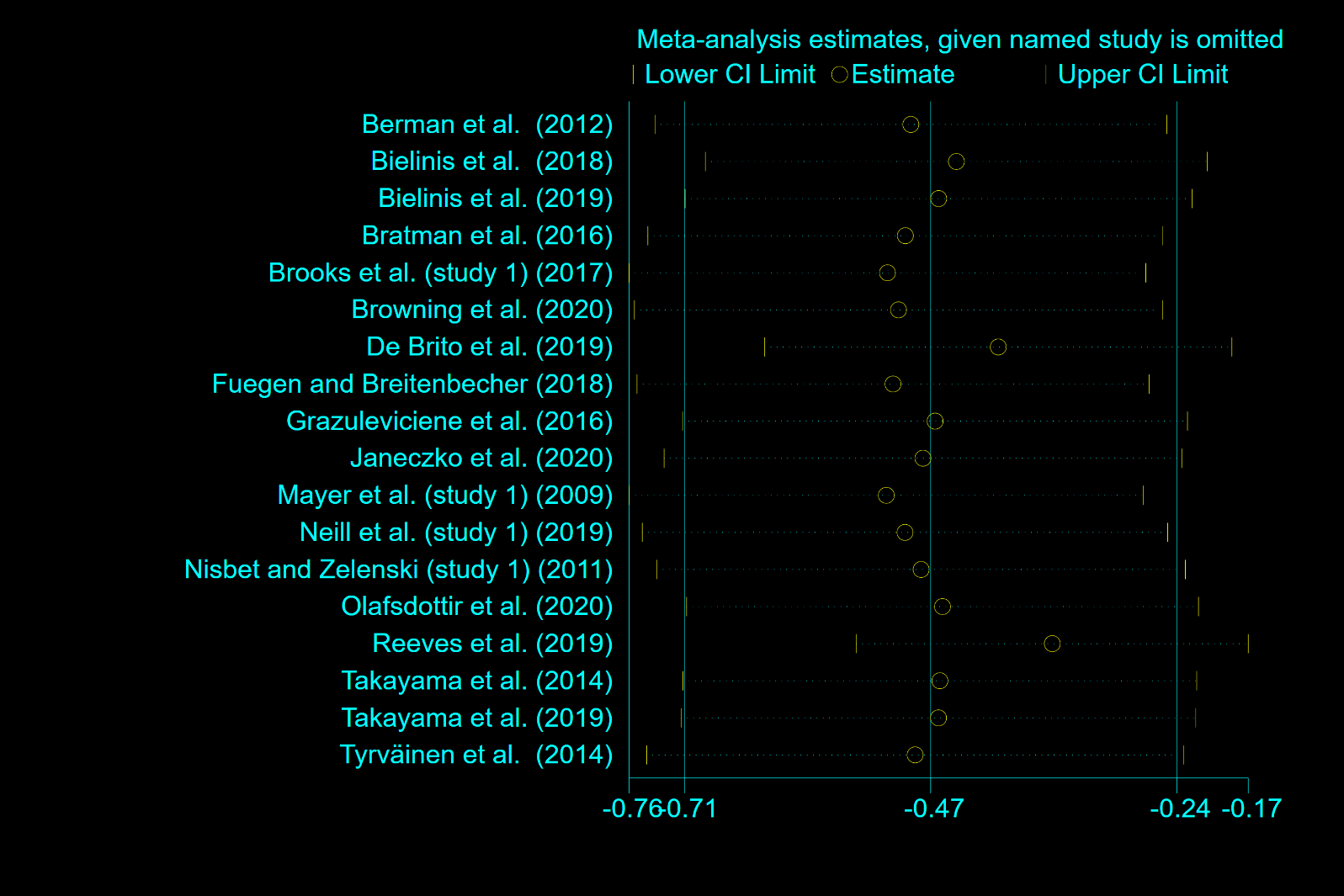


**Fig.S2 Sensitivity analysis of the association between natural exposure and negative affect.**

**Tab.S5 Meta regression model with most important moderators of positive affect.**

| Variable | Coef. | SE | z | *p* | CI | *tau²* |
| --- | --- | --- | --- | --- | --- | --- |
| Study Region |  |  |  |  |  | 0.1754 |
| North America | 0.0718 | 0.2192 | 0.33 | 0.743 | [-0.358, 0.501] |  |
| Europe | 0.0906 | 0.2278 | 0.40 | 0.691 | [-0.356, 0.537] |  |
| Aisa | -0.4024 | 0.3399 | -1.18 | 0.236 | [-1.069, 0.264] |  |
| Study Design |  |  |  |  |  | 0.0940 |
| Within-subject | 0.3686 | 0.3533 | 1.04 | 0.297 | [-0.324, 1.061] |  |
| Between-subject | 0.5277 | 0.2121 | 2.49 | **0.013** | [0.112, 0.943] |  |
| Mixed factorial design | -0.5782 | 0.1790 | -3.23 | **0.001** | [-0.929, -0.227] |  |
| Mean age | 0.0056 | 0.0094 | 0.59 | 0.553 | [-0.013, 0.023] | 0.1663 |
| Sample size | -0.0020 | 0.0024 | -0.81 | 0.417 | [-0.007, 0.003] | 0.1731 |
| Female (%) | 0.0023 | 0.0044 | 0.53 | 0.597 | [-0.006, 0.011] | 0.1696 |
| Total exposure Time | 0.0036 | 0.0072 | 0.49 | 0.623 | [-0.011, 0.018] | 0.1782 |
| Type of natural environment |  |  |  |  |  | 0.2158 |
| Biodiverse area | 0.0679 | 0.2778 | 0.24 | 0.807 | [-0.477, 0.612] |  |
| Forest | 0.1111 | 0.2273 | 0.49 | 0.625 | [-0.334, 0.557] |  |
| Urban park | -0.1747 | 0.2377 | -0.74 | 0.462 | [0.6406, 0.2911] |  |
| University campus | -0.0681 | 0.3524 | -0.19 | 0.847 | [-0.759, 0.623] |  |
| Type of built environment |  |  |  |  |  | 0.1712 |
| City-street | -0.0639 | 0.2288 | -0.28 | 0.780 | [-0.512, 0.384] |  |
| Building site | -0.2268 | 0.2209 | -1.03 | 0.305 | [-0.659, 0.206] |  |
| Indoor | 0.5712 | 0.2836 | 2.01 | 0.044 | [0.016, 1.127] |  |
| Active category |  |  |  |  |  | 0.1946 |
| Active | -0.0028 | 0.2202 | -0.01 | 0.990 | [-0.434, 0.429] |  |
| Passive | -0.0007 | 0.2344 | -0.00 | 0.997 | [-0.460, 0.458] |  |
| Mixed | 0.0070 | 0.3142 | 0.02 | 0.982 | [-0.609, 0.623] |  |

**Tab.S6 Meta regression model with most important moderators of negative affect.**

| Variable | Coef. | SE | z | *p* | CI | *tau²* |
| --- | --- | --- | --- | --- | --- | --- |
| Study Region |  |  |  |  |  | 0.2498 |
| North America | 0.5729 | 0.2534 | 2.26 | **0.024** | [0.076, 1.069] |  |
| Europe | -0.5443 | 0.2748 | -1.98 | **0.048** | [-1.083, -0.006] |  |
| Aisa | -0.1734 | 0.3768 | -0.46 | 0.645 | [-0.912, 0.565] |  |
| Study Design |  |  |  |  |  | 0.3455 |
| Within-subject | -0.5137 | 0.4641 | -1.11 | 0.268 | [-1.423, 0.395] |  |
| Between-subject | 0.0155 | 0.3704 | 0.04 | 0.967 | [-0.711, 0.742] |  |
| Mixed factorial design | 0.2221 | 0.3177 | 0.70 | 0.485 | [-0.401, 0.845] |  |
| Mean age | -0.0246 | 0.0124 | -2.05 | **0.040** | [-0.048, -0.001] | 0.2675 |
| Sample size | 0.0072 | 0.0029 | 2.55 | **0.011** | [0.002, 0.013] | 0.2185 |
| Female (%) | 0.0008 | 0.0065 | 0.13 | 0.901 | [-0.013, 0.015] | 0.4078 |
| Total exposure Time | -0.0085 | 0.0099 | -0.85 | 0.407 | [-0.029, 0.013] | 0.3193 |
| Type of natural environment |  |  |  |  |  | 0.3258 |
| Biodiverse area | -0.5953 | 0.3423 | -1.74 | 0.082 | [-1.266, 0.076] |  |
| Forest | -0.1043 | 0.3049 | -0.34 | 0.732 | [-0.702, 0.493] |  |
| Urban park | 0.4373 | 0.3425 | 1.28 | 0.202 | [-0.234, 1.108] |  |
| University campus | 0.3875 | 0.4567 | 0.85 | 0.396 | [-0.508, 1.283] |  |
| Type of built environment |  |  |  |  |  | 0.3022 |
| City-street | -0.2908 | 0.2932 | -0.99 | 0.321 | [-0.865, 0.283] |  |
| Building site | 0.2883 | 0.3069 | 0.94 | 0.348 | [-0.313, 0.889 |  |
| Indoor | 0.5628 | 0.4235 | 1.33 | 0.184 | [-0.267, 1.392] |  |
| Active category |  |  |  |  |  | 0.3585 |
| Active | 0.2091 | 0.2945 | 0.71 | 0.478 | [-0.368, 0.786] |  |
| Passive | -0.0702 | 0.4070 | -0.17 | 0.863 | [-0.868, 0.727] |  |
| Mixed | -0.1889 | 0.3116 | -0.61 | 0.544 | [-0.799, 0.421] |  |

**Fig.S3 Galbraith plot for positive affect studies (n=20).**

**Fig.S4 Galbraith plot for negative affect studies (n=18)**.


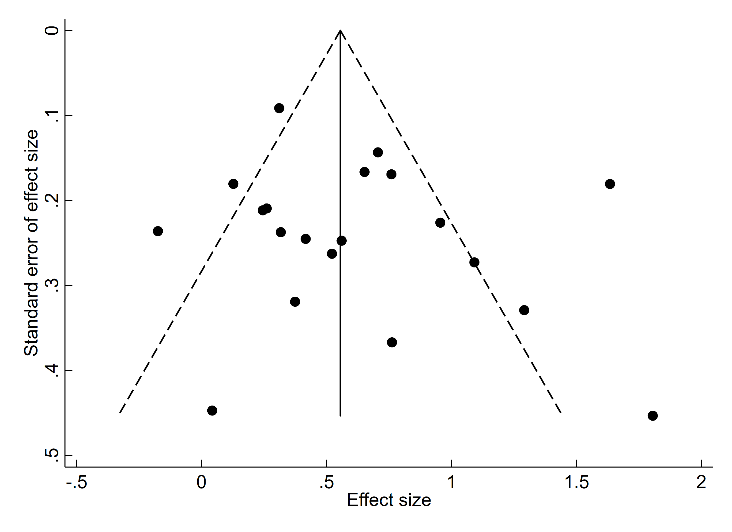

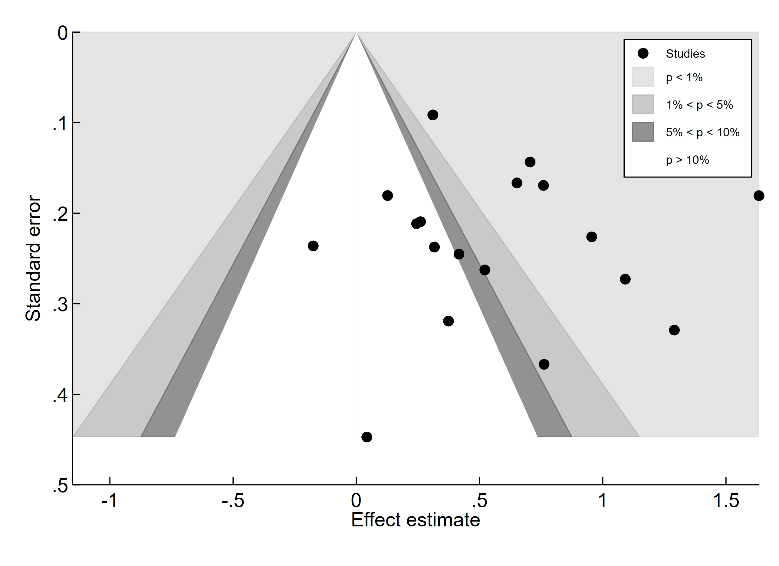


**Fig.S5 Funnel plot to access potential publication bias of the positive affect studies (n=18).**


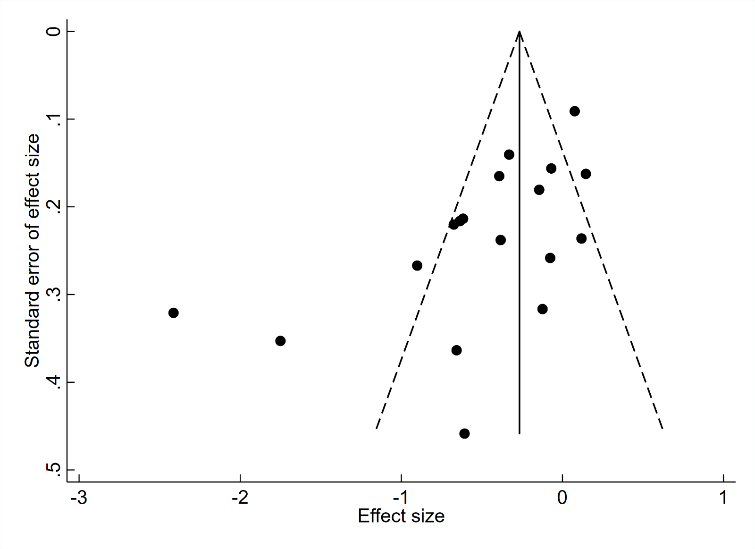

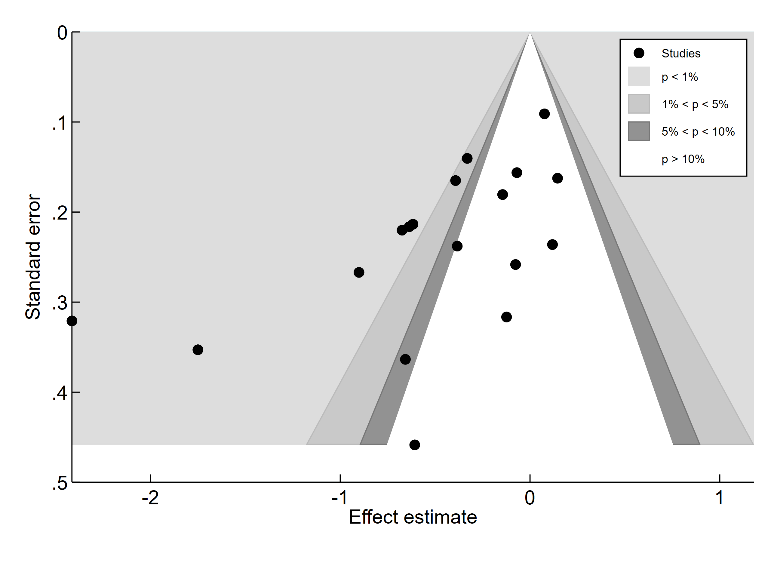


**Fig.S6 Funnel plot to access potential publication bias of the negative affect studies (n=18).**

**Tab.S7 GRADE evaluation of pooled evidence for associations between natural exposure and positive and negative affect.**

| **Quality assessment** | | | | | **No of participants** | | **Effect size** | **Quality** |
| --- | --- | --- | --- | --- | --- | --- | --- | --- |
| **Risk of bias** | **Inconsistency** | **Indirectness** | **Imprecision** | **Publication bias** | **Natural** | **Built** |  |  |
| **Positive Affect** | | | | | | | | |
| Serious | Serious | Direct | No | No | 1101 | 1044 | 0.61 (0.41, 0.81) | Low |
| **Negative Affect** | | | | | | | | |
| Serious | Serious | Direct | No | Yes | 1053 | 996 | -0.47 (-0.71, -0.24) | Very Low |
